# Compatible versus annual stand-growth models for thinned Chinese fir plantations

**DOI:** 10.3389/fpls.2025.1698282

**Published:** 2025-11-14

**Authors:** Yihang Jiang, Quang V. Cao, Jun Chen, Jianguo Zhang, Xiongqing Zhang

**Affiliations:** 1 State Key Laboratory of Efficient Production of Forest Resources, Key Laboratory of Tree Breeding and Cultivation of the National Forestry and Grassland Administration, Research Institute of Forestry, Chinese Academy of Forestry, Beijing, China; 2 Collaborative Innovation Center of Sustainable Forestry in Southern China, Nanjing Forestry University, Nanjing, China; 3 School of Renewable Natural Resources, Louisiana State University Agricultural Center, Baton Rouge, LA, United States

**Keywords:** stand survival, stand basal area, annual growth, compatible growth model, thinned stands

## Abstract

**Introduction:**

Chinese fir (Cunninghamia lanceolata) plantations are a major timber resource in subtropical China, where thinning strongly affects stand growth and yield forecasting. Reliable models are needed to balance short-term post-thinning responsiveness with long-term path consistency. This study compares two stand-level systems—Compatible and Annual (periodic-annual) Growth models—to evaluate their performance across thinning regimes and forecast horizons.

**Methods:**

Data were derived from a 40-year thinning trial in southern China. Stand survival and basal area were modeled for thinned and unthinned stands using two model classes: (i) a Compatible system ensuring algebraic path consistency and (ii) an Annual system emphasizing short-interval responsiveness. Each was fitted with two data structures: consecutive (non-overlapping) and all possible (overlapping) growth pairs. Parameters were estimated using Seemingly Unrelated Regressions (SUR) and validated through threefold cross-validation for short (2–4 yr), medium (6–8 yr), and long (≥10 yr) horizons.

**Results:**

In unthinned stands, Annual models with all growth pairs achieved the best short-term accuracy, while Compatible models performed best at medium and long horizons. In thinned stands, Annual models most accurately captured short-term survival, whereas Compatible models yielded superior basal area predictions at short to medium horizons. All-pairs estimation generally improved precision, though consecutive pairs reduced bias in short-term post-thinning basal area.

**Discussion:**

Model behavior revealed a trade-off between responsiveness and path consistency. Annual systems are advantageous immediately after thinning, while Compatible systems provide stable long-term projections. For management of C. lanceolata plantations, we recommend Annual + All-pairs for short-term forecasting and Compatible + All-pairs for medium-to-long horizons, with recalibration every 6–8 years to maintain predictive reliability.

## 1 Introduction

Thinning is one of the most influential interventions in plantation silviculture. By reducing stand density and altering canopy structure, thinning modifies microclimate and resource competition, often accelerate the growth of residual trees—but responses are strongly context-dependent, varying with timing, intensity, stand development, and site quality (Allen et al., 2021; Aussenac, 2000; Li et al., 2021; Ma et al., 2010; Rambo and North, 2009). For management, this variability creates a modelling challenge: operational decisions require stand-level projections of survival and basal area (BA) that remain reliable immediately after treatment and over subsequent decades. Long-term thinning trials, with irregular measurement intervals and treatment sequences, therefore call for models that (i) preserve coherence across varying step lengths and treatment histories (path consistency) and (ii) remain responsive to short-interval post-thinning dynamics.

These requirements align with two established stand-level modelling traditions. Compatible growth-and-yield systems impose algebraic consistency between increment and state equations; integrating growth recovers the yield function and, in principle, produces path-invariant projections across varying step lengths and through sequences of thinning (Bailey and Clutter, 1974; Burkhart and Tomé, 2012; Clutter, 1963; Clutter et al., 1983; Vanclay, 1994). Such systems are attractive for medium- to long-term planning in managed stands because they track trajectories smoothly through multiple interventions. In contrast, annual growth models estimate short-interval changes (e.g., survival, basal area) from the current state and modifiers (density, site, climate, explicit thinning effects). Their structural flexibility can capture the sharp, near-term responses that follow thinning, though the absence of algebraic path invariance can allow bias to accumulate over long horizons (Ochi and Cao, 2003; Weiskittel et al., 2011). In thinning trials, these contrasts set up an explicit trade-off between long-horizon path consistency and short-horizon responsiveness.

A second critical choice concerns how to structure longitudinal data for parameter estimation. Using all-possible (overlapping) growth pairs leverages variable interval lengths, increases effective sample size, and can better capture nonlinearities. On the other hand, using consecutive (non-overlapping) pairs simplifies dependence structures and may curb error compounding when simulating far beyond the calibration window. Evidence from site-index and stand-growth studies shows that the performance of these data structures depends on model class, dynamic regime, and forecast horizon (Cieszewski and Strub, 2007; Wang et al., 2007). Because thinning interacts with density-regulated processes—classically framed by size–density relationships (Reineke, 1933)—the optimal pairing of model class and data structure may itself depend on thinning status and time since treatment.

Chinese fir (*Cunninghamia lanceolata*) plantations, a keystone species comprising over 25% of China’s plantation area, holds significant economic and ecological value. Long-term density-management trials document survival and basal area responses across a range of thinning intensities (Li et al., 2021; Tang et al., 2016; Zhang et al., 2020). In this study, we develop and evaluate two stand-level systems—a Compatible Growth system and an Annual Growth system—for projecting survival and basal area in thinned and unthinned stands, each fitted using Consecutive Growth Pairs and All Growth Pairs designs. We ask, within a single, rigorously monitored thinning trial, whether model class and data structuring interact with thinning status and forecast horizon in ways that matter for practice.

Guided by theory and prior comparisons, we hypothesize that:

- Annual models will outperform at short horizons, when post-thinning responses are strongest, whereas Compatible systems will outperform at medium–long horizons due to path consistency (Ochi and Cao, 2003; Weiskittel et al., 2011);
- All Growth Pairs will enhance precision and short-term accuracy by exploiting variable intervals, whereas Consecutive pairs may reduce error propagation in long-term projections (Cieszewski and Strub, 2007; Wang et al., 2007); and
- performance rankings will shift systematically with thinning status and horizon, reflecting immediate microclimatic and competitive changes after thinning versus later structural equilibration (Aussenac, 2000; del Río et al., 2017; Rambo and North, 2009).

Rather than proposing universally applicable yield equations from a single-site trial, our goal is a ***methods-focused case study*** that distills transferable decision rules for selecting model class and data organization in plantation thinning applications, complementing insights from long-term experiments across species and regions (del Río et al., 2017; Pretzsch, 2019; Vanclay, 1994).

## 2 Materials and methods

### 2.1 Materials

The data used in this research were obtained from Chinese fir plantations established in 1981 at Nianzhu Forest Farm, located in Fenyi City, Jiangxi Province, southern China (27.82°N, 114.68°E). This region is characterized by a subtropical maritime monsoon climate, with an average annual precipitation of 1656 mm, a mean annual temperature of 16.8 °C, and an average annual evaporation of 503 mm. The soil is laterite, known for its high humus content.

The experimental design followed a randomized block layout with five planting densities: A) 2 m × 3 m (1,667 trees/ha); B) 2 m × 1.5 m (3,333 trees/ha); C) 2 m × 1 m (5,000 trees/ha); D) 1 m × 1.5 m (6,667 trees/ha); E) 1 m × 1 m (10,000 trees/ha). Each density treatment was replicated three times, resulting in 15 experimental plots (each measuring 20 × 30 m), labeled A1-A3, B1-B3, C1-C3, D1-D3, and E1-E3. Each plot was bordered by a buffer zone of two rows of trees. Tree growth parameters, including tree height (H) and diameter at breast height (DBH, 1.3 m), were recorded biennially from 1986 to 2002.

**Figure 1:**
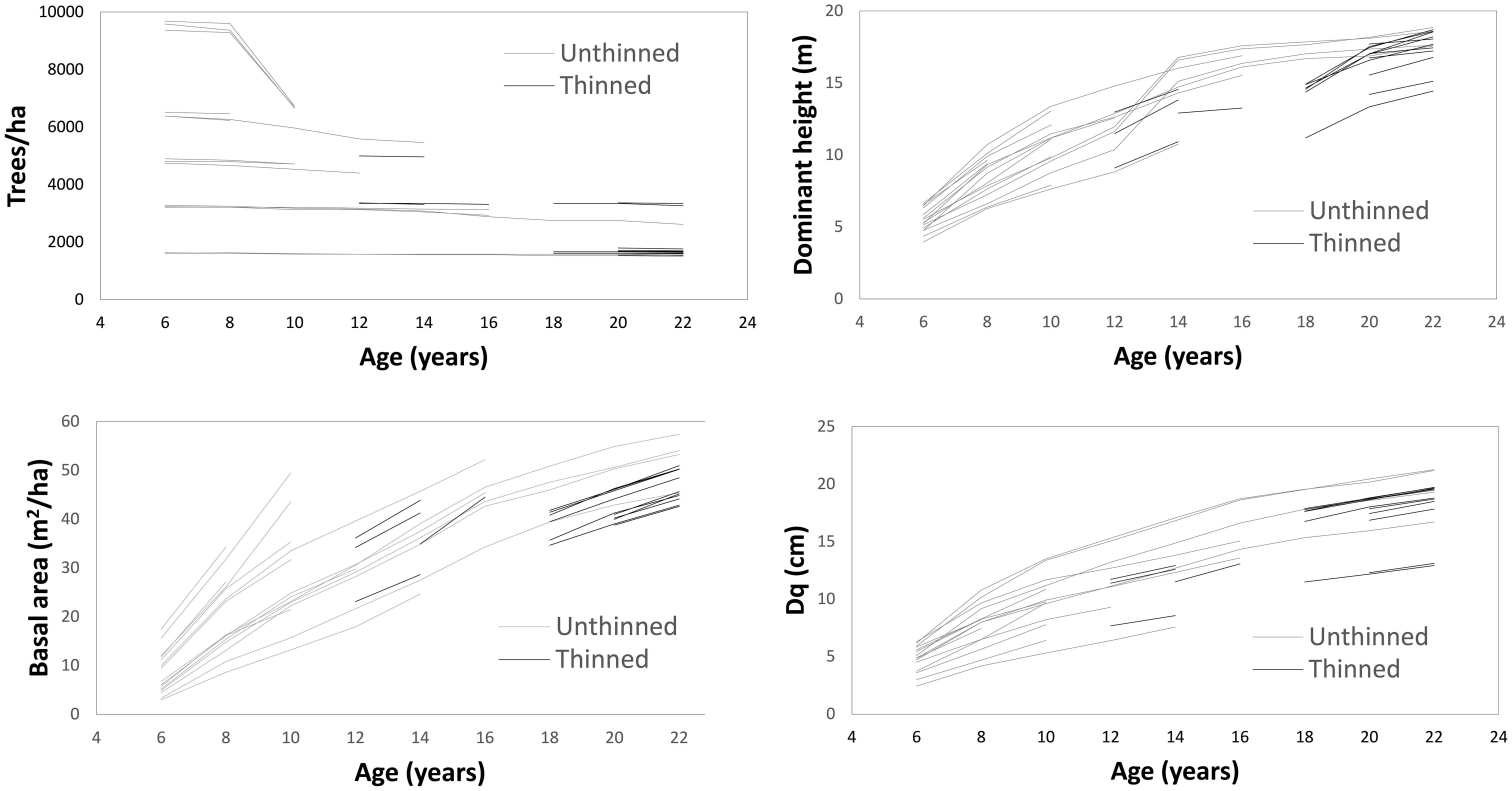
Growth over time for number of trees and basal area per ha, dominant height, and quadratic mean diameter (*Dq*).

Thinning operations were conducted from below, reducing most stands to a predefined lower planting density. As stands reached this density threshold at different ages due to growth variation across plots, thinning timing varied accordingly. Detailed thinning histories for each plot are provided in **Table 1**, while **Figure 1** illustrates temporal trends in trees per ha, basal area, dominant height, and quadratic mean diameter (Dq) for thinned and unthinned stands.

**Table 1:** Thinning history for each plot.

Planting density	Rep	Thinning	Age of thinning	After thinning	
(trees/ha)			(years)	#trees/ha	m^2^/ha
1667	1, 2, 3	None			
3333	1	None			
	2	First	18	1550	38.78
	3	First	18	1683	33.53
5000	1	First	14	3383	36.35
		Second	18	1717	36.00
	2	First	12	3333	36.19
		Second	16	1667	35.66
	3	First	12	3350	39.96
		Second	16	1738	37.42
6667	1	First	16	4967	32.65
		Second	18	3367	33.99
	2	First	10	4983	36.03
		Second	12	3350	36.15
		Third	16	1687	36.34
	3	First	10	4954	46.75
		Second	12	3350	38.69
		Third	16	1700	37.80
10000	1	First	12	5000	23.11
		Second	16	3367	30.18
	2	First	12	3400	27.23
		Second	18	1800	33.92
	3	First	12	3367	34.21
		Second	16	1667	32.37

### 2.2 Methods

#### 2.2.1 Two data types for parameter estimation

Two approaches were implemented to generate data used for estimating the regression coefficients of the models. In the first approach, *consecutive growth pairs* at successive time points were used to estimate the parameters. For example, if a plot was measured at ages 6, 8, 10, and 12, the resulting growth intervals would be 6–8 years, 8-10, and 10-12. The second approach considered *all possible growth pairs* in a forward time sequence. Using the same example, the growth intervals would include 2–6 years, 6-10, 6-12, 8-10, 8-12, and 10-12.

#### 2.2.2 Two types of models

##### 2.2.2.1 Compatible growth models

Clutter (1963) defined compatibility as the requirement that the yield model be obtainable through mathematical integration of the growth model. Additionally, a compatible growth model should be step-invariant, meaning the final outcome remains the same whether the stand is grown directly from age *A*_1_ to age *A*_3_ or indirectly through age *A*_2_ (Peng, 2000).

For thinned/unthined stands, stand survival of the current year was assumed to be a proportion of the previous year’s stand density.

(1a) $${\hat{N}}_{2i}=\{\begin{array}{cc} exp\left[b_{1}+\left\{ln\left(N_{1i}\right)-b_{1}\right\}{\left(\frac{A_{2i}}{A_{1i}}\right)}^{b_{2}}\right] & ,\text{ if unthinned}\\ b_{3}^{\left(A_{2i}-A_{1i}\right)}N_{1i} & ,\;\;\text{if thinned} \end{array}$$

The function used to project stand basal area for both thinned and unthinned stands followed the model developed by Bailey and Clutter (1974):

(1b) $${\hat{B}}_{2i}=exp\left[c_{1}+\left\{ln\left(B_{1i}\right)-c_{1}\right\}{\left(\frac{A_{2i}}{A_{1i}}\right)}^{c_{2}}\right]$$

where *N*_1_*_i_* and *B*_1_*_i_* are, respectively, number of trees/ha and stand basal area (m^2^/ha) for plot *i* at time 1, ${\hat{N}}_{2i}$ and ${\hat{B}}_{2i}$ are stand survival and basal area at time 2, respectively, and *A*_1_*_i_* and *A*_2_*_i_* are stand age in years at the beginning and end of the growing period. The coefficients *b’s* and *c’s* are parameters estimated for the survival and basal area submodels, respectively, in the Annual model.

The parameters of Equations 1a and 1b, which together form a system for simultaneously predicting *N* (number of trees per ha) and *B* (basal area per ha), were estimated using the Seemingly Unrelated Regressions (SUR) method (SAS proc MODEL, SAS Institute Inc. 2004).

##### 2.2.2.2 Annual growth models

Dominant height at time (t+1), ${\hat{H}}_{i,\;t+1}$, was predicted from dominant height at time *t*, *H_i,t_*, by use of Bailey and Clutter’s (1974) equation (Equations 2a). Equations to project stand survival and basal area were modified from Chen et al. (2024).

(2a) $${\hat{H}}_{i,\;t+1}=exp\left[a_{1}+\left\{ln\left(H_{i,\;t}\right)-a_{1}\right\}{\left(\frac{A_{i,t+1}}{A_{it}}\right)}^{c_{2}}\right]$$

(2b) $${\hat{N}}_{i,\;t+1}=\{\begin{array}{cc} N_{i,\;t}-exp\left[b_{0}+b_{1}RS_{i,\;t}+b_{2}A_{i,\;t}+b_{3}\text{ln}\left(H_{i,\;t}\right)\right] & ,\text{ if unthinned}\\ b_{4}N_{i,\;t} & ,\;\;\text{if thinned} \end{array}$$

(2c) $${\hat{B}}_{i,\;t+1}=B_{i,\;t}-exp\left[c_{0}+c_{1}RS_{i,\;t}+c_{2}A_{i,\;t}\right]$$

where N*_i_*_,t_, B*_i_*_,t_, and RS*_i,t_* are number of trees/ha, stand basal area (m^2^/ha), and relative spacing (RS*_i,t_*=(10000/*N_i,t_*)^0.5/*H_i,t_*) for plot *i*, respectively, at time *t*, ${\hat{N}}_{i,t+1}$ and ${\hat{B}}_{i,t+1}$ are, respectively, stand survival and basal area at time (*t* + 1).

The SUR method was again employed to estimate parameters *b*’s and *c*’s of Equations 2b and 2c (Pinheiro and Bates, 2000).

#### 2.2.3 Evaluation

A three-fold cross-validation method was used to evaluate four alternatives (2 models × 2 data types). This process involved creating three data groups, each comprising a fit data set (including two replications from each treatment) and a validation data set (consisting of the remaining replication). Parameter estimates obtained from the fit data were used to predict stand attributes of the validation data. Predicted values from the combined data were subsequently used to calculate the following evaluation statistics (Equations 3a–c):

(3a) $$Mean\;difference:MD=\sum_{i}\left(y_{2i}-{\hat{y}}_{2i}\right)/m$$

(3b) $$Mean\;absolute\;difference:MAD=\sum_{i}\left|y_{2i}-{\hat{y}}_{2i}\right|/m$$

(3c) $$Fit\;index:FI=1-\sum_{i}{\left(y_{2i}-{\hat{y}}_{2i}\right)}^{2}/\sum_{i}\left(y_{2i}-{\bar{y}}_{2}\right)$$

where *m* is number of growth intervals; $y_{2i}$ and ${\hat{y}}_{2i}$ are, respectively, observed and predicted values of *N* or *B* of plot *i* at the end of the growth period; and ${\bar{y}}_{2}$ is average of $y_{2i}$.

The relative position of each method for growth prediction was determined by use of the relative rank system, introduced by Poudel and Cao (2013). In this approach, the best and worst methods among *k* methods (where *k* = 4, representing 2 models × 2 data types) were assigned relative ranks of 1 and k, respectively. The remaining methods received real-number ranks between 1 and *k* were given as relative ranks for the remaining methods. Because this ranking system accounts for both the magnitude and order of each evaluation statistic, it provides more information than the traditional ordinal ranking method (Poudel and Cao, 2013).

## 3 Results

### 3.1 Parameter estimates

Parameter estimates for both the Compatible and Annual Growth models were stable across grouping schemes and data types, and all coefficients were statistically significant at the 5% level (**Table 2**). This consistency indicates that the functional forms are well identified for both survival and basal area dynamics under Chinese fir management.

**Table 2:** Parameter estimates for the growth models. All estimates are significant at the 5% level.

Growth pairs	Model	Parameter	Group 1	Group 2	Group 3	All
Consecutive	Compatible	*b*_1_	8.0794	8.0297	8.0605	8.0683
		*b*_2_	-0.4644	-0.4600	-0.4618	-0.4857
		*b*_3_	0.9959	0.9966	0.9906	0.9963
		*c*_1_	4.3370	4.4935	4.4889	4.4511
		*c*_2_	-1.8052	-1.4567	-1.4097	-1.5262
	Annual growth	*a*_1_	3.2951	3.2524	3.2884	3.2834
		*a*_2_	-1.1794	-1.1704	-1.1037	-1.1445
		*b*_0_	57.5192	98.7311	63.9822	94.6156
		*b*_1_	-188.225	-306.790	-195.922	-289.617
		*b*_2_	0.7119	1.0745	0.3719	1.0285
		*b*_3_	-15.4598	-28.9893	-16.0587	-27.8865
		*b*_4_	0.9958	0.9957	0.9954	0.9956
		*c*_0_	3.0596	3.0950	2.4364	2.92497
		*c*_1_	-0.1131	-0.1088	-0.0791	-0.1025
		*c*_2_	-2.0041	-2.7134	-1.2059	-2.1168
All	Compatible	*b*_1_	7.5331	7.4490	7.4981	7.4978
		*b*_2_	-0.3262	-0.2749	-0.2811	-0.2918
		*b*_3_	0.9957	0.9961	0.9949	0.9960
		*c*_1_	4.3492	4.5244	4.5533	4.4860
		*c*_2_	-1.5885	-1.2929	-1.2529	-1.3447
	Annual growth	*a*_1_	3.2361	3.2039	3.2389	3.2298
		*a*_2_	-1.3486	-1.3079	-1.2444	-1.2872
		*b*_0_	57.6206	40.4247	46.6826	63.4825
		*b*_1_	-183.396	-104.042	-124.828	-187.667
		*b*_2_	0.9034	0.2570	0.2947	0.5622
		*b*_3_	-16.5821	-10.4191	-12.1188	-17.3996
		*b*_4_	0.9960	0.9963	0.9971	0.9962
		*c*_0_	2.6376	2.4751	2.2262	2.4325
		*c*_1_	-0.0944	-0.0808	-0.06872	-0.0806
		*c*_2_	-1.4131	-1.6785	-1.1887	-1.3928

### 3.2 Model performance in unthinned stands

Validation against independent data showed clear, horizon-specific performance patterns (**Tables 3**, **4**). For stand survival, the least-biased predictions (lowest MD) in the short term came from the Annual Growth model fitted with consecutive growth pairs (MD = 1.62), while the highest overall accuracy for short-term survival—lowest MAD and highest FI—was achieved by the Annual Growth model fitted with All Growth Pairs (MAD = 118.28; FI = 0.9832). In medium- and long-term projections, the Compatible model with All Growth Pairs consistently produced the best accuracy (lowest MAD; highest FI), outperforming the other three method–data combinations (**Table 3**).

**Table 3:** Evaluation statistics 1/ (and their relative ranks) for stand survival prediction of *unthinned stands*.

Growth pairs	Model	MD		MAD		FI	
Short-term projection (2 - 4 years) – n = 107							
Consecutive	Compatible	-92.43	(4.00)	221.25	(4.00)	0.9552	(3.88)
	Annual growth	***1.62***	***(1.00)***	141.20	(1.67)	0.9655	(2.82)
All	Compatible	14.26	(1.42)	204.41	(3.51)	0.9540	(4.00)
	Annual growth	8.51	(1.23)	***118.28***	***(1.00)***	***0.9832***	***(1.00)***
Medium-term projection (6 - 8 years) – n = 58							
Consecutive	Compatible	-228.16	(4.00)	257.76	(4.00)	0.9262	(2.82)
	Annual growth	***16.14***	***(1.00)***	132.49	(1.02)	0.9397	(2.21)
All	Compatible	22.55	(1.09)	***131.51***	***(1.00)***	***0.9667***	***(1.00)***
	Annual growth	67.65	(1.73)	156.80	(1.60)	0.8998	(4.00)
Long-term projection (10 years or more) – n = 42							
Consecutive	Compatible	-480.41	(4.00)	480.41	(4.00)	0.1999	(4.00)
	Annual growth	***46.57***	***(1.00)***	159.76	(1.39)	0.7709	(1.71)
All	Compatible	-84.22	(1.26)	***111.30***	***(1.00)***	***0.9485***	***(1.00)***
	Annual growth	110.35	(1.44)	203.21	(1.75)	0.5641	(2.54)

For each projection length, a bold, italic number denotes the best method based on each evaluation statistic.

1/ *MD*, mean difference (number of trees/ha); *MAD*, mean absolute difference; *FI*, fit index.

**Table 4:** Evaluation statistics 1/ (and their relative ranks) for stand basal area prediction of *unthinned stands*.

Growth pairs	Model	MD		MAD		FI	
Short-term projection (2 - 4 years) – n = 107							
Consecutive	Compatible	-0.8173	(3.32)	2.4238	(2.39)	0.9297	(1.79)
	Annual growth	-0.9899	(4.00)	2.6874	(4.00)	0.9019	(4.00)
All	Compatible	0.2652	(1.13)	***2.1952***	***(1.00)***	***0.9397***	***(1.00)***
	Annual growth	***0.2320***	***(1.00)***	2.4525	(2.57)	0.9163	(2.86)
Medium-term projection (6 - 8 years) – n = 58							
Consecutive	Compatible	-2.9780	(4.00)	3.2135	(3.80)	0.7814	(2.82)
	Annual growth	-2.2619	(3.06)	3.3012	(4.00)	0.6969	(4.00)
All	Compatible	-0.7664	(1.10)	***1.9621***	***(1.00)***	***0.9126***	***(1.00)***
	Annual growth	***-0.6891***	***(1.00)***	2.5058	(2.22)	0.8452	(1.94)
Long-term projection (10 years or more) – n = 42							
Consecutive	Compatible	-3.7234	(4.00)	3.9822	(4.00)	0.1601	(4.00)
	Annual growth	-2.4325	(2.95)	3.1737	(2.57)	0.4120	(2.68)
All	Compatible	-0.5567	(1.43)	2.5031	(1.39)	0.6882	(1.23)
	Annual growth	***-0.0211***	***(1.00)***	***2.2819***	***(1.00)***	***0.7316***	***(1.00)***

For each projection length, a bold, italic number denotes the best method based on each evaluation statistic.

1/ *MD*, mean difference (m^2^/ha); *MAD*, mean absolute difference; *FI*, fit index.

For basal area, short-term accuracy was highest for the Compatible model with All Growth Pairs (lowest MAD = 2.1952; highest FI = 0.9397), whereas the Annual model with All Growth Pairs minimized bias (MD = 0.2320). In the medium term, the Compatible model with All Growth Pairs again yielded the best MAD and FI, while the Annual model with All Growth Pairs had the lowest MD. In the long term, the Annual model with All Growth Pairs dominated across all three statistics (lowest MD and MAD; highest FI), indicating strong persistence of predictive skill at extended horizons (**Table 4**).

### 3.3 Model performance in thinned stands-

In thinned stands, the Annual Growth model provided the most accurate survival predictions. Short-term projections were best when parameters were estimated with All Growth Pairs (lowest MD and MAD; highest FI), while medium-term projections favored parameters from consecutive pairs (again leading on all three metrics) (**Table 5**). For basal area, short-term accuracy was highest for the Compatible model with consecutive pairs (best MD, MAD, and FI). Over the medium term, performance depended on the criterion: the Annual model with All Growth Pairs minimized MD and MAD, whereas the Compatible model with All Growth Pairs achieved the highest FI (**Table 6**).

**Table 5:** Evaluation statistics 1/ (and their relative ranks) for stand survival prediction of *thinned stands*.

Growth pairs	Model	MD		MAD		FI	
Short-term projection (2 - 4 years) – n = 55							
Consecutive	Compatible	4.46	(2.97)	21.41	(4.00)	0.9991	(4.00)
	Annual growth	4.72	(3.10)	19.97	(2.04)	0.9993	(1.00)
All	Compatible	6.76	(4.00)	21.32	(3.87)	0.9992	(2.50)
	Annual growth	***-0.06***	***(1.00)***	***19.21***	***(1.00)***	***0.9993***	***(1.00)***
Medium-term projection (6 - 8 years) – n = 6							
Consecutive	Compatible	-31.23	(4.00)	31.23	(4.00)	0.9955	(4.00)
	Annual growth	***-4.40***	***(1.00)***	***17.18***	***(1.00)***	***0.9991***	***(1.00)***
All	Compatible	-30.89	(3.96)	31.08	(3.97)	0.9961	(3.50)
	Annual growth	-15.54	(2.25)	22.22	(2.08)	0.9984	(1.58)

For each projection length, a bold, italic number denotes the best method based on each evaluation statistic.

1/ *MD*, mean difference (m^2^/ha); *MAD*, mean absolute difference; *FI*, fit index.

**Table 6:** Evaluation statistics 1/ (and their relative ranks) for stand basal area prediction of *thinned stands*.

Growth pairs	Model	MD		MAD		FI	
Short-term projection (2 - 4 years) – n = 55							
Consecutive	Compatible	***-0.1030***	***(1.00)***	***0.9036***	***(1.00)***	***0.9246***	***(1.00)***
	Annual growth	0.6797	(3.78)	1.1969	(4.00)	0.8827	(4.00)
All	Compatible	0.3420	(2.15)	0.9582	(1.56)	0.9121	(1.89)
	Annual growth	0.7256	(4.00)	1.1119	(3.13)	0.8902	(3.46)
Medium-term projection (6 - 8 years) – n = 6							
Consecutive	Compatible	-1.3456	(4.00)	1.3456	(4.00)	0.7486	(4.00)
	Annual growth	0.5190	(1.48)	0.9551	(2.40)	0.8387	(2.52)
All	Compatible	-0.5578	(1.60)	0.7192	(1.43)	***0.9314***	***(1.00)***
	Annual growth	***0.3619***	***(1.00)***	***0.6130***	***(1.00)***	0.9305	(1.01)

For each projection length, a bold, italic number denotes the best method based on each evaluation statistic.

1/ *MD*, mean difference (m^2^/ha); *MAD*, mean absolute difference; *FI*, fit index.

The integrated rank analysis (**Table 7**) summarizes these patterns. In unthinned stands, the All Growth Pairs approach ranked first overall at all horizons, with the Annual model leading in the short term and the Compatible model taking over in the medium and long term. In thinned stands, the combination of the Annual Growth model with All Growth Pairs ranked best in the short term, and the Annual model remained top-ranked into the medium term (with the consecutive-pairs variant a close second), underscoring the robustness of the Annual formulation under active thinning.

**Table 7:** Overall rank for each method based on the rank total.

Treatment	Growth pairs	Model	Sum of the ranks		Total	Overall
			based on N	based on B		rank
Unthinned	***Short-term projection (2 - 4 years)***					
	Consecutive	Compatible	11.88	7.50	19.38	4.00
		Annual growth	5.49	12.00	17.49	3.42
	All	Compatible	8.93	3.13	12.06	1.74
		Annual growth	3.23	6.43	***9.65***	***1.00***
	***Medium-term projection (6 - 8 years)***					
	Consecutive	Compatible	10.82	10.63	21.44	4.00
		Annual growth	4.23	11.06	15.30	2.79
	All	Compatible	3.09	3.10	***6.19***	***1.00***
		Annual growth	7.33	5.16	12.49	2.24
	***Long-term projection (10 years or more)***					
	Consecutive	Compatible	12.00	12.00	24.00	4.00
		Annual growth	4.11	8.21	12.31	1.90
	All	Compatible	3.26	4.05	***7.31***	***1.00***
		Annual growth	5.73	3.00	8.73	1.25
Thinned	***Short-term projection (2 - 4 years)***					
	Consecutive	Compatible	10.97	3.00	13.97	1.26
		Annual growth	6.12	11.78	17.90	4.00
	All	Compatible	10.37	5.61	15.98	2.66
		Annual growth	3.00	10.59	***13.59***	***1.00***
	***Medium-term projection (6 - 8 years)***					
	Consecutive	Compatible	12.00	12.00	24.00	4.00
		Annual growth	3.00	6.40	9.40	1.10
	All	Compatible	11.43	4.03	15.46	2.30
		Annual growth	5.91	3.01	***8.92***	***1.00***

A bold, italic number denotes the best method.

### 3.4 Thinning simulation

To illustrate the modeling approach, we simulated the growth trajectory of a representative plot (plot B2), incorporating a thinning event.

**Figure 2:**
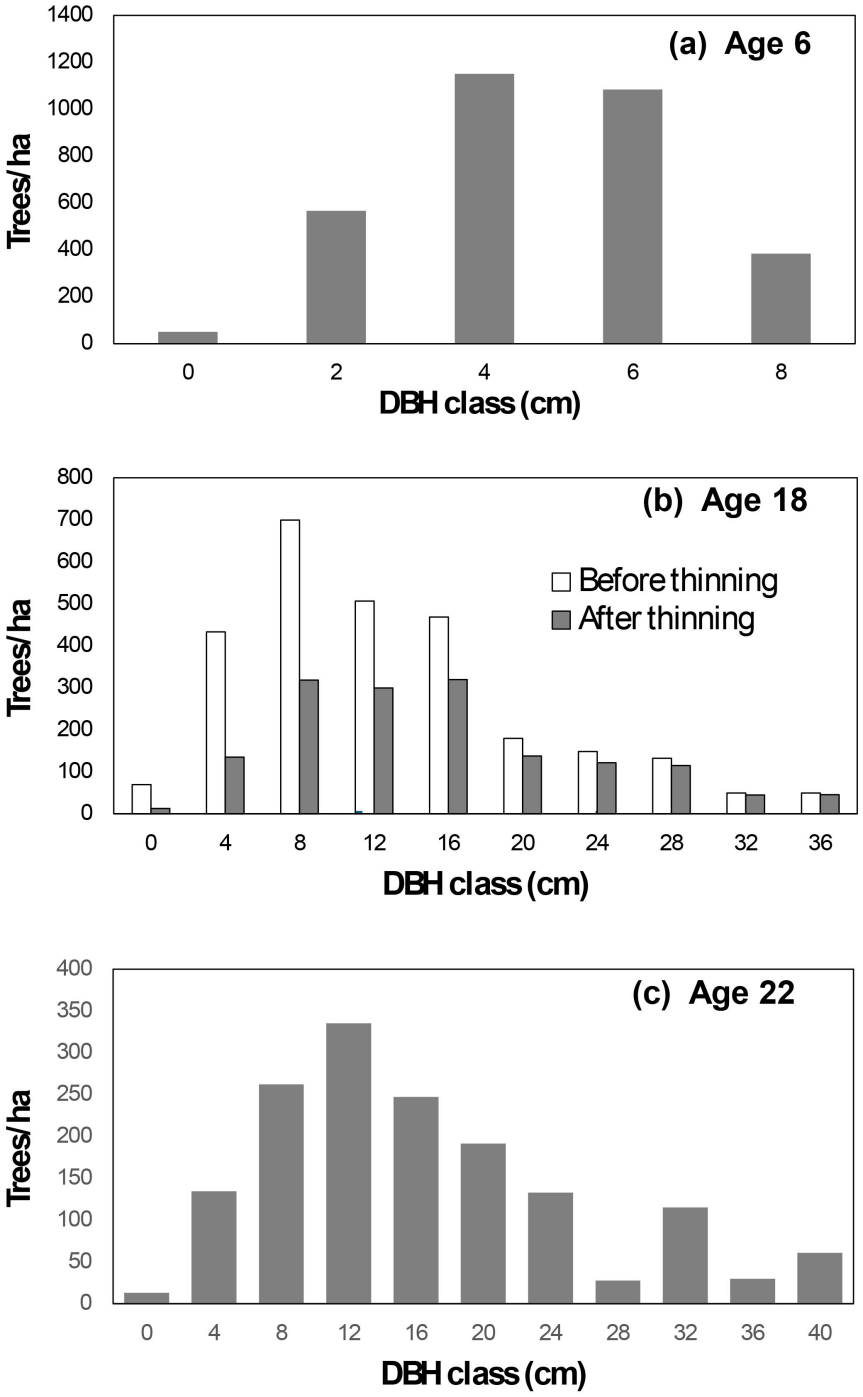
Diameter distribution for plot B2 at age 6 **(a)**. Simulated diameter distributions for this plot are presented for ages 18 **(b)** and 22 **(c)**.

***Initial conditions:*** The simulation began with observed data from plot B2 at age 6, where the stand comprised 3,233 trees/ha and a stand basal area of 6.01 m²/ha. Tree diameters followed a unimodal distribution (**Figure 2a**).
***Growth to age 18:*** Using Compatible Growth Models (Equations 1a, 2b) and parameters derived from all possible growth pairs (**Table 2**), the stand was projected to age 18. At this stage, the predicted stand survival and basal area were 2,755 trees/ha and 45.71 m^2^/ha, respectively.
***Tree list at age 18 (Before Thinning):*** The tree list at age 6 was updated to age 18 by use of a tree-level growth model derived from the stand growth model (Cao, 2019). The survival probability of tree *j* in plot *i* (*p_ij_*) and tree diameter at the end of the growth period ( ${\hat{d}}_{2ij}$) was calculated based on the initial tree diameter *d*_1_*_ij_* (cm) at age 6 Equations 4a, b:

(4a) $$p_{ij}=1-exp\left[\alpha_{1}\left(d_{1ij}-0.95Dmin_{1i}\right)\right]$$

(4b) $${\hat{d}}_{2ij}=d_{1ij}\;exp\left(\alpha_{2}d_{1ij}\right)$$

where *Dmin*_1_*_i_* represents the minimum diameter (cm) in plot *i* at the beginning of the growth period, and *a*_1_ and *a*_2_ are coefficients ensuring consistency with stand-level outputs Equations 5a, b:

, and (5a) $${\hat{N}}_{2i}=\sum_{j=1}^{n_{1i}}p_{ij}/s$$

(5b) $${\hat{B}}_{2i}=\sum_{j=1}^{n_{1i}}Kp_{ij}{\hat{d}}_{2ij}^{2}/s$$

Here, ${\hat{N}}_{2i}$ = 2755 trees/ha and ${\hat{B}}_{2i}$ 45.71 m^2^/ha are, respectively, predicted stand survival and basal area (m^2^/ha) of plot *i* at the end of the growing period (age 18). The coefficient K = π/40000, and *s* represents the plot size in hectares. The resulting diameter distribution is illustrated in **Figure 2b**.

***Tree list at age 18 (After Thinning):*** At age 18, the stand was thinned to 1,550 trees/ha. Following thinning, the stand basal area was reduced to 36.18 m^2^/ha with the post-thinning diameter distribution shown in **Figure 2b**.
***Growth to age 22:*** The stand was further projected to age 22, maintaining 1,550 trees/ha and reaching a basal area of 44.73 m^2^/ha. The tree list was updated using the same methods, with the resulting diameter distribution depicted in **Figure 2c**.

**Figure 3:**
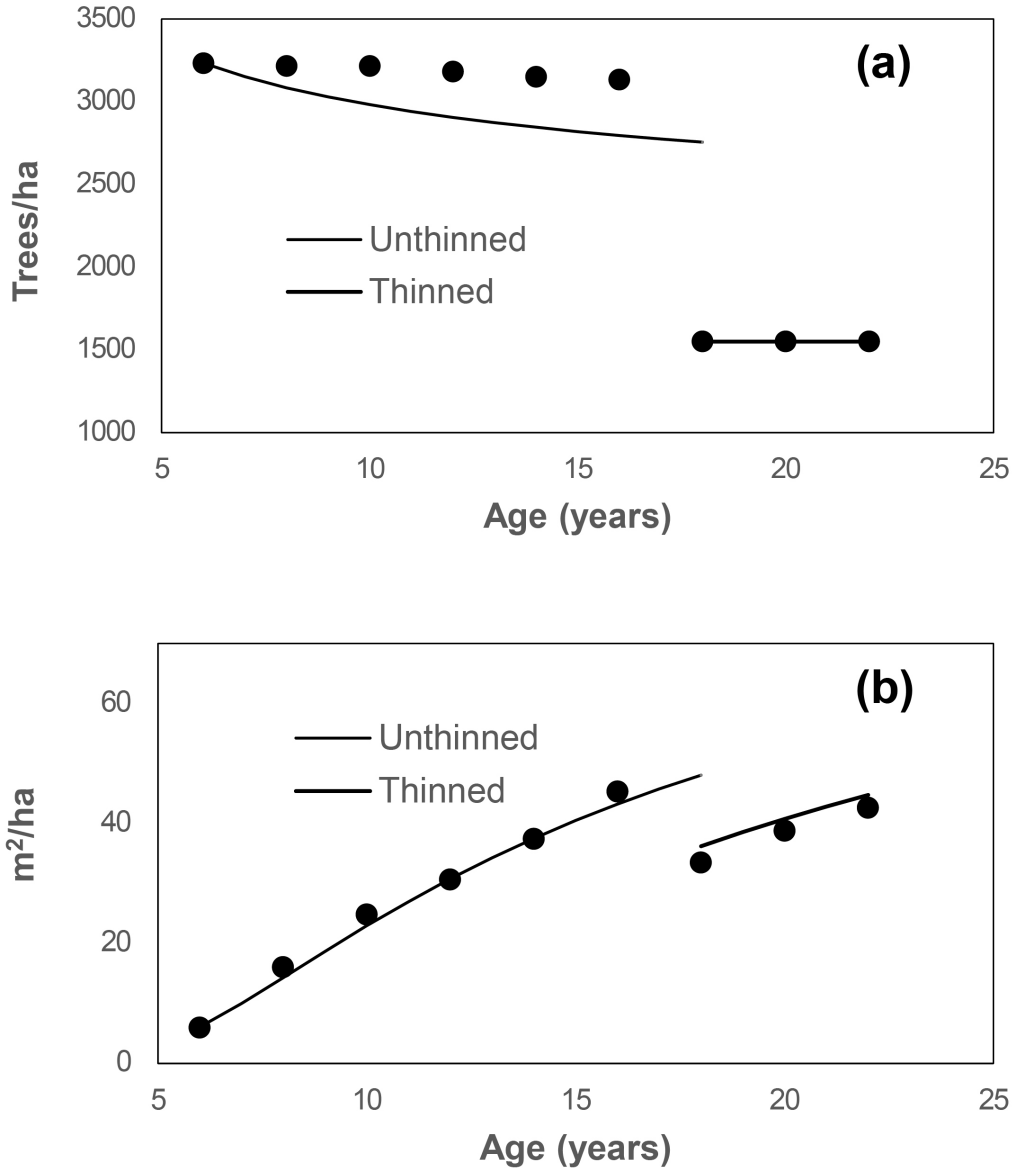
Simulation of stand survival **(a)** and basal area **(b)** from age 6 to 22 for plot B2, initially with 3233 trees/ha and a stand basal area of 6.01 m²/ha at age 6. At age 18, the stand was thinned to 1550 trees/ha. Circles represent observed values for this plot.

**Figure 3** presents the observed and predicted values for stand survival and basal area from age 6 to age 22, including the thinning event at age 18. While stand survival was slightly underestimated, the stand basal area was modeled well for both unthinned and thinned scenarios.

## 4 Discussion

Chinese fir plantations are intensively managed with thinning, yet forecasting stand dynamics after treatment still hinges on a fundamental trade-off: preserving path consistency across variable step lengths versus capturing short-interval responses to canopy opening and density change (Aussenac, 2000; del Río et al., 2017; Rambo and North, 2009). By contrasting a compatible growth-and-yield system with an annual (periodic-annual) system, each calibrated with either consecutive or all possible growth pairs, we show that model class and data structuring interact with thinning status and forecast horizon in ways that matter operationally for both survival and stand basal area.

### 4.1 Survival and basal area after thinning

Annual survival fractions in thinned stands were close to unity (0.991–0.997), indicating low background mortality under the observed treatment intensities and site conditions (**Table 2**). Even with high mean survival, thinning can transiently elevate risk via wind exposure, heat stress, or mechanical damage—mechanisms widely reported across conifers (Bose et al., 2018; Powers et al., 2010; Pretzsch, 2020). This aligns with classic survival formulations that treat mortality as a small, density- and treatment-conditioned deviation from a high baseline (Bailey et al., 1985). From a management perspective, such “small” deviations matter most in the first years after treatment, when compounding over short steps can shift yield trajectories (Allen et al., 2021; Li et al., 2021; Ma et al., 2010). Short-horizon projections benefit from structures that explicitly model annual change.

For stand basal area, the immediate post-thinning balance between removed stems and compensatory growth by residual trees proved decisive. Short-term BA predictions were most accurate with the Compatible model calibrated on consecutive pairs (**Table 6**), indicating that a path-consistent aggregation of increment into state performs best for this near-term equilibrium. At medium horizons, method rankings depended on the evaluation statistic (**Table 6**), consistent with theory: as stands re-equilibrate, BA dynamics are governed by rapid diameter growth in released trees and the gradual rebuilding of leaf area and competition (Aussenac, 2000; del Río et al., 2017; Pretzsch, 2019; Rambo and North, 2009).

In unthinned stands, BA accuracy favored Compatible + All-pairs at short–medium horizons and shifted toward Annual + All-pairs at longer horizons (**Table 4**), underscoring the broader trade-off between path consistency and short-interval responsiveness (Burkhart and Tomé, 2012; Weiskittel et al., 2011). In thinned stands, consecutive pairs can curb error propagation for short-term BA, whereas the optimal model–data pairing shifts with time since treatment as structural recovery progresses.

### 4.2 All growth-pair estimation: benefits and caveats

Using all possible (overlapping) growth pairs exploited variable interval lengths and consistently improved tracking of non-linear trajectories—for both survival and BA—especially in unthinned stands. Short-term survival accuracy favored Annual + All-pairs, whereas Compatible + All-pairs led at medium horizons. For BA, Compatible + All-pairs performed best at short–medium horizons, with Annual + All-pairs regaining an edge at longer horizons. These outcomes match prior comparisons: annual systems excel at capturing short-interval variability, while compatible systems propagate coherently across variable step lengths and interventions (Burkhart and Tomé, 2012; Ochi and Cao, 2003; Weiskittel et al., 2011).

Methodologically, overlapping pairs increase effective sample size and help identify curvature (Cieszewski and Strub, 2007; Corral-Rivas et al., 2004), but they also introduce dependence among observations and between state and increment equations. Mixed-effects formulations can help reduce serial correlation, but fitting multi-equation (SUR-type) systems with random effects remains challenging in standard software. In our case, the pragmatic approach—fitting Equations 1a and 1b separately and treating parameters b_1_ and c_1_ as plot-specific random effects—led to convergence failures and was therefore abandoned. Although the SUR framework accounts for cross-equation correlation between survival and basal area, it cannot fully eliminate temporal dependence among overlapping intervals. Future work could address this issue using hierarchical or state-space approaches.

### 4.3 Horizon-specific performance in thinned and unthinned stands

**Unthinned stands**: A clear horizon effect emerged. Over 2–4 years, Annual + All-pairs gave the highest survival accuracy and competitive BA errors; over 6–8 years, Compatible + All-pairs led for both survival and BA; beyond ~10 years, overall accuracy declined and dispersion widened, with Annual + All-pairs regaining the edge for BA but not achieving short-term fit levels (**Tables 3**, **4**). The integrated ranking (**Table 7**) consolidates this: All-pairs is the preferred estimation strategy in unthinned stands, with Annual best at short horizons and Compatible best from medium onward.

**Thinned stands**: Short-term survival was most accurate with Annual + All-pairs, whereas medium-term survival favored Annual + consecutive (**Table 5**). For BA, Compatible + consecutive led in the short term, while medium-term BA performance depended on the evaluation statistic (**Table 6**). These divergences reflect the shift from immediate microclimatic/competitive responses toward structural equilibration as stands recover from thinning (Aussenac, 2000; del Río et al., 2017; Rambo and North, 2009) and align with density-regulated processes framed by size–density relationships (Reineke, 1933).

Taken together, the survival–BA patterns confirm: (i) annual formulations are preferable immediately after thinning; (ii) All-pairs generally enhances precision—particularly in unthinned stands—while consecutive pairs can be advantageous for short-term BA after thinning; and (iii) rankings shift systematically with thinning status and horizon.

### 4.4 Management implications for Chinese fir plantations

Two practical decision rules follow that distinguish survival and BA:

***(1) Choose estimation strategy first, then model class.***

- **Unthinned stands:** Prefer All-pairs irrespective of model. Use Annual for short-horizon survival/BA and Compatible for medium–long horizons.
- **Thinned stands:** For survival, use Annual + All-pairs in the short term and Annual + consecutive in the medium term. For BA, use Compatible + consecutive in the short term; reassess at medium horizons based on the target criterion (bias vs. dispersion vs. fit index).

***(2) Plan medium-term decision cycles (~6–8 years) with routine recalibration.***

This interval retains high predictive skill for both survival and BA while limiting structural drift; projections extending far beyond the calibration window should be refreshed as new inventory data arrive (Clutter et al., 1983; Kangas, 1999; Vanclay, 1994; Vanclay and Skovsgaard, 1997).

More broadly, our findings reinforce the complementary roles of compatible and annual systems in managed plantations: compatible models provide path-consistent, parsimonious forecasts across variable step lengths—well suited to medium–long horizons and treatment sequences—whereas annual models deliver responsiveness where managers need it most, immediately after thinning. This division of labor echoes comparative evidence across conifers for both survival and growth/BA responses (del Río et al., 2017; Mäkinen and Isomäki, 2004; Pretzsch, 2019).

### 4.5 Limitations and next steps

Our single-trial design constrains ecological generality, and we did not implement fully joint (SUR) mixed-effects estimation or explicit climate modifiers. BA inferences may also be sensitive to measurement protocol (e.g., plot size, edge corrections) and the translation from tree-level growth to stand-level BA. Future work should test these decision rules across site-quality and thinning-intensity gradients (Li et al., 2021), incorporate climate/soil covariates where data permit (Allen et al., 2015), and evaluate hierarchical, state-space formulations that deliver distributional forecasts and explicit uncertainty propagation for both survival and BA (Weiskittel et al., 2011). Such extensions would further align operational projections with the dual requirements laid out in the Introduction: path consistency through treatment sequences and responsiveness to short-interval post-thinning dynamics.

## 5 Conclusion

This study demonstrates that reliable projections of survival and stand basal area in Chinese fir hinge on matching model class to horizon and thinning status, and on selecting an estimation data structure suited to the stand dynamics. In unthinned stands, using All Growth Pairs generally improves accuracy; Annual Growth models perform best at short horizons, whereas Compatible Growth models gain the advantage from medium to long horizons. Following thinning, Annual Growth models capture the near-term survival response more effectively, whereas short-term basal area is represented more accurately by a path-consistent Compatible Growth model fitted with consecutive pairs, as stands move toward re-equilibration.

In practice, begin by selecting the estimation scheme—All Growth Pairs for unthinned stands, or consecutive pairs when short-term post-thinning basal area is the priority—then choose Annual or Compatible to match the decision horizon. Recalibrate every 6–8-years to limit drift and sustain forecast quality. Applied this way, the two approaches are complementary: Annual Growth offers short-interval responsiveness around interventions, while Compatible Growth delivers coherent trajectories for medium- to long-term planning and timber-yield forecasting.

## Data Availability

The original contributions presented in the study are included in the article/supplementary material, further inquiries can be directed to the corresponding author/s.

## References

[B1] Allen C. D. Breshears D. D. McDowell N. G. (2015). On underestimation of global vulnerability to tree mortality and forest die-off from hotter drought in the Anthropocene. Ecosphere 6, 1–55. doi: 10.1890/ES15-00203.1

[B2] Allen M. Brunner A. Antón-Fernández C. Astrup R. (2021). The relationship between volume increment and stand density in Norway spruce plantations. Forestry 94, 151–165. doi: 10.1093/forestry/cpaa020

[B3] Aussenac G. (2000). Interactions between forest stands and microclimate: Ecophysiological aspects and consequences for silviculture. Ann. For. Sci. 57, 287–301. doi: 10.1051/forest:2000119

[B4] Bailey R. L. Borders B. E. Ware K. D. Jones E. P. (1985). A compatible model relating slash pine plantation survival to density, age, site index, and thinning. For. Sci. 31, 180–189. doi: 10.1093/forestscience/31.1.180

[B5] Bailey R. L. Clutter J. L. (1974). Base-age invariant polymorphic site curves. For. Sci. 20, 155–159. doi: 10.1093/forestscience/20.2.155

[B6] Bose A. K. Weiskittel A. R. Kuehne C. Wagner R. G. Turnblom E. Burkhart H. E. (2018). Tree-level growth and survival following commercial thinning of four major softwood species in North America. For. Ecol. Manage. 427, 355–364. doi: 10.1016/j.foreco.2018.06.019

[B7] Burkhart H. E. Tomé M. (2012). Modeling Forest Trees and Stands (Dordrecht: Springer). doi: 10.1007/978-90-481-3170-9

[B8] Cao Q. V. (2019). A method to derive a tree survival model from any existing stand survival model. Can. J. For. Res. 49, 1598–1603. doi: 10.1139/cjfr-2019-0171

[B9] Cieszewski C. J. Strub M. (2007). Parameter estimation of base-age invariant site index models: Which data structure to use?—A discussion. For. Sci. 53, 552–555. doi: 10.1093/forestscience/53.5.552

[B10] Chen H. Cao Q. V. Jiang Y. Hu Y. Zhang J. Zhang X. (2024). Modeling stand- and tree-level growth of Chinese fir plantations. Can. J. For. Res. 54, 686–697. doi: 10.1139/cjfr-2023-0195

[B11] Clutter J. L. (1963). Compatible growth and yield models for loblolly pine. For. Sci. 9, 354–371. doi: 10.1093/forestscience/9.3.354

[B12] Clutter J. L. Fortson J. C. Pienaar L. V. Brister G. H. Bailey R. L. (1983). Timber Management: A Quantitative Approach (New York: Wiley).

[B13] Corral-Rivas J. J. Álvarez-González J. G. Ruiz-González A. D. von Gadow K. (2004). Compatible height and site index models for five pine species in El Salto, Durango (Mexico). For. Ecol. Manag. 201, 145–160. doi: 10.1016/j.foreco.2004.05.060

[B14] del Río M. Bravo-Oviedo A. Ruiz-Peinado R. Onrubia R. (2017). A review of thinning effects on Scots pine stands. For. Syst. 26, eR03. doi: 10.5424/fs/2017262-11325

[B15] Kangas A. S. (1999). Methods for assessing uncertainty of growth and yield predictions. Can. J. For. Res. 29, 1357–1364. doi: 10.1139/x99-100

[B16] Li Y. Xu J. Wang H. Nong Y. Sun G. Yu S. . (2021). Long-term effects of thinning and mixing on stand spatial structure: a case study of Chinese fir plantations. iForest 14, 113–121. doi: 10.3832/ifor3489-014

[B17] Ma S. Concilio A. Oakley B. North M. Chen J. (2010). Spatial variability in microclimate in a mixed-conifer forest before and after thinning and burning treatments. For. Ecol. Manage. 259, 904–915. doi: 10.1016/j.foreco.2009.11.030

[B18] Mäkinen H. Isomäki A. (2004). Thinning intensity and long-term changes in increment and stem form of Scots pine trees. For. Ecol. Manage. 203, 21–34. doi: 10.1016/j.foreco.2004.07.028

[B19] Ochi N. Cao Q. V. (2003). A comparison of compatible and annual growth models. For. Sci. 49, 285–290. doi: 10.1093/forestscience/49.2.285

[B20] Peng C. H. (2000). Growth and yield models for uneven-aged stands: Past, present and future. For. Ecol. Manage. 132, 259–279. doi: 10.1016/S0378-1127(99)00229-7

[B21] Pinheiro J. C. Bates D. M. (2000). Mixed-Effects Models in S and S-PLUS (New York: Springer). doi: 10.1007/b98882

[B22] Poudel K. P. Cao Q. V. (2013). Evaluation of methods to predict Weibull parameters to characterize diameter distributions. For. Sci. 59, 243–252. doi: 10.5849/forsci.12-001

[B23] Powers M. D. Palik B. J. Bradford J. B. Fraver S. Webster C. R. (2010). Thinning method and intensity influence long-term mortality trends in a red pine forest. For. Ecol. Manage. 260, 1138–1148. doi: 10.1016/j.foreco.2010.07.002

[B24] Pretzsch H. (2019). Maintenance of long-term experiments for unique insights into forest growth dynamics and trends: review and perspectives. Eur. J. For. Res. 138, 165–185. doi: 10.1007/s10342-018-1151-y

[B25] Pretzsch H. (2020). Density and growth of forest stands revisited: Effect of the temporal scale of observation, site quality and thinning. For. Ecol. Manage. 460, 117879. doi: 10.1016/j.foreco.2020.117879

[B26] Rambo T. R. North M. P. (2009). Canopy microclimate response to pattern and density of thinning in a Sierra Nevada mixed-conifer forest. For. Ecol. Manage. 257, 435–442. doi: 10.1016/j.foreco.2008.09.029

[B27] Reineke L. H. (1933). Perfecting a stand-density index for even-aged forests. J. Agric. Res. 46, 627–638.

[B28] SAS Institute Inc. (2004). SAS/ETS 9.1 User’s Guide. SAS Institute Inc., Cary, NC. 2416p.

[B29] Tang X. Zhao D. Kane M. (2016). Development of stand density management diagrams for Chinese fir plantations. Forestry 89, 36–45. doi: 10.1093/forestry/cpv024

[B30] Vanclay J. K. (1994). Modelling Forest Growth and Yield: Applications to Mixed Tropical Forests (Wallingford, UK: CAB International).

[B31] Vanclay J. K. Skovsgaard J. P. (1997). Evaluating forest growth models. Ecol. Model. 98, 1–12. doi: 10.1016/S0304-3800(96)01932-1

[B32] Wang M. Borders B. E. Zhao D. (2007). Parameter estimation of base-age invariant site index models: Which data structure to use? For. Sci. 53, 541–551. doi: 10.1093/forestscience/53.5.541

[B33] Weiskittel A. R. Hann D. W. Kershaw J. A. Jr. Vanclay J. K. (2011). Forest Growth and Yield Modeling (Chichester: Wiley-Blackwell). doi: 10.1002/9781119998518

[B34] Zhang X. Cao Q. V. Wang H. Duan A. Zhang J. (2020). Projecting stand survival and basal area based on a self-thinning model for Chinese fir plantations. For. Sci. 66, 361–370. doi: 10.1093/forsci/fxz086

